# Oral Administration of Piperine as Curative and Prophylaxis Reduces Parasitaemia in *Plasmodium berghei* ANKA-Infected Mice

**DOI:** 10.1155/2022/5721449

**Published:** 2022-03-22

**Authors:** Shafia Khairani, Nisa Fauziah, Hesti Lina Wiraswati, Ramdan Panigoro, Endang Yuni Setyowati, Afiat Berbudi

**Affiliations:** ^1^Doctoral Program, Medical Science, Faculty of Medicine, Padjadjaran University, Bandung, Indonesia; ^2^Veterinary Medicine Program, Faculty of Medicine, Padjadjaran University, Bandung, Indonesia; ^3^Department of Biomedical Sciences, Parasitology Division, Faculty of Medicine, Padjadjaran University, Bandung, Indonesia; ^4^Department of Biomedical Sciences, Biochemistry and Molecular Biology Division, Faculty of Medicine, Padjadjaran University, Bandung, Indonesia

## Abstract

Malaria remains a public health problem and a leading cause of death worldwide. Consequently, the discovery of novel agents, including substances from medicinal plants, is urgently needed. *Piper nigrum* has long been used by the community in the treatment of the symptoms of malaria. In a previous study, *Piper nigrum* was demonstrated to exhibit promising antiplasmodial activity against *Plasmodium falciparum* 3D7 and INDO strains. The aim of this study was to further investigate the antimalarial activity (curative and prophylactic) of piperine (a major isolated constituent of *Piper nigrum)* in *Plasmodium berghei* ANKA-infected mice. Piperine 10, 20, and 40 mg/kg body weight (bw), artesunate 5 mg/kg bw, and DMSO were administered orally for four days to different groups of Swiss Webster mice. Then, mice were monitored for parasitaemia, body weight, rectal temperature, survival rate, and clinical parameters. Piperine 40 mg/kg bw in curative and prophylactic tests had the maximum parasitaemia chemosuppression of 79.21% and 58.8% (*p* < 0.05), respectively, with a significant effect on the survival rate compared with control animals. In the curative test, piperine 40 mg/kg bw reduced the mean clinical score compared with the control group. Additionally, piperine showed an ability to protect organs (lungs, liver, spleen, and kidneys) from some damage in a dose-dependent manner. This study can be used as a basis for further discovery of novel chemotherapeutic or chemoprophylactic compounds.

## 1. Introduction

Recent advances in our understanding of the biology and pathogenesis of the *Plasmodium* parasite are currently unsatisfactory. Malaria is still recognized as a highly contagious disease, widespread in the tropics and subtropics, and is still one of the most significant causes of human death [1]. Malaria was responsible for 241 million cases in 2020, with an estimated 627,000 deaths worldwide [2]. Gradually, several exciting breakthroughs have been made in the prevention and treatment of malaria, including mosquito-repellent sprays, insecticide-treated bed nets, medicines, and vaccines [3]. Recently, the malaria vaccine RTS, S was introduced for children in areas of moderate and high *Plasmodium falciparum* transmission; however, it does not provide absolute protection from the disease and required multiple boosters [4, 5].

In the absence of an effective vaccine, artemisinin-based combination therapies (ACTs) are the only frontline therapeutic to treat malaria disease. In fact, drug resistance is inevitable due to the emergence of more virulent strains of *Plasmodium falciparum* and other pathogens, such as *Plasmodium knowlesi* (the cause of severe malaria in humans with a mortality rate of 2%) [6]. According to the WHO malaria eradication agenda, the discovery of novel chemical entities that exhibit multistage activity against the parasite, good therapeutic index, and low toxicity is in high demand, as well as novel and multiple mechanisms of action [7, 8].

Plants and plant extracts have shown potential effectiveness in treating various diseases, such as malaria. Most of them are also known to have a wide margin of safety [9, 10]. Nature has gifted some of the plants around us with potential effects against *Plasmodium* parasites [11–13]. Interestingly, halofantrine, quinine, chloroquine, mefloquine, and artemisinin (the currently available malaria drug) are of plant origin [14, 15].

Piperine is one of the main active components isolated from *P. nigrum* [16]. *Piper nigrum* has been widely used by traditional South Indian healers to treat common colds, intermittent fevers, asthma, colic pains, cholera, diarrhoea, as well as malaria [17–20]. The ethyl acetate extract of *Piper nigrum* has been described as having promising antiplasmodial activity, with IC_50_ values of 12.5 and 12.0 g/mL in *Plasmodium falciparum* 3D7 and INDO strains, respectively. It also showed low toxicity (TC_50_ = 87.0 g/ml) and a significant therapeutic index [17].

*Plasmodium* is a unicellular protozoan with a complex life cycle in different hosts. Briefly, an infected female *Anopheles* mosquito bites a human host, injects ∼100 *Plasmodium* sporozoites into the dermis, and enters the bloodstream. The sporozoites reach the liver through the bloodstream or lymphatic system, and thereafter, the sporozoites invade, traverse, develop, and multiply in hepatocytes (exoerythrocytic stage). Furthermore, sporozoites undergo schizogony and then produce tens of thousands of merozoites and release them into the bloodstream (erythrocytic stage) [21]. Each stage of the life cycle has advantages as a drug target but also has associated risks. Currently, most of the available antimalarial compounds are stage-specific, for example, quinine and chloroquine, well known as schizonticidal drugs, prompting us to test new compounds against multiple stages for a broader spectrum of effects. To the best of our knowledge, this is the first study that investigates the curative and prophylactic activities of piperine (a major isolated constituent of *Piper nigrum*) using *Plasmodium berghei* ANKA-infected mice.

## 2. Materials and Methods

### 2.1. Mouse and Parasite Strains

Swiss Webster 8–12-week-old male mice weighing 25–30 g were used for this study. Mice were acclimated for one week to a 12-h/12-h light/dark cycle, fed commercial pellets, and supplied with water *ad libitum*. The *Plasmodium berghei* ANKA used in this study was obtained from the Malaria Laboratory of the Faculty of Pharmacy, Jenderal Achmad Yani University.

### 2.2. Drug Treatment Protocol

**Figure 1:**
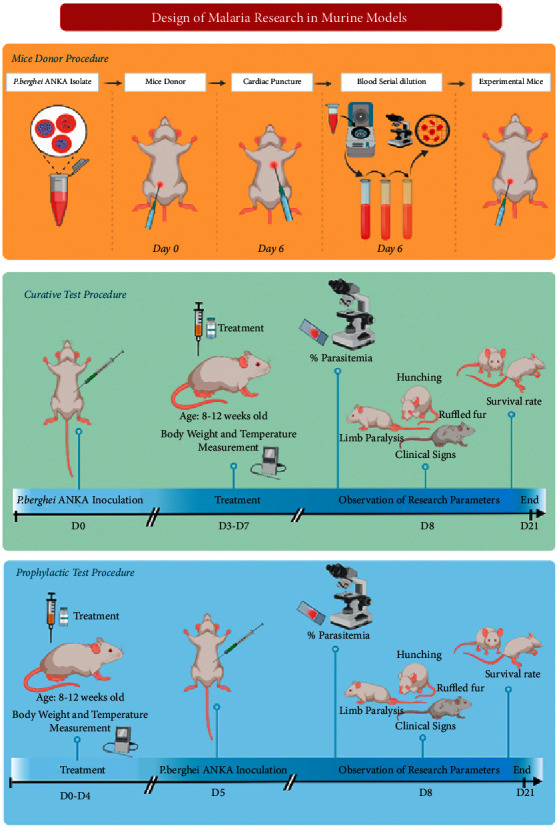
Malaria research design using murine models.

Piperine (catalogue no. P49007, ≥97%) and artesunate (catalogue no. A3731) were purchased from Sigma-Aldrich Inc. All compounds were dissolved in dimethyl sulfoxide (DMSO) and given orally by gavage to mice. Parasitaemia levels of the donor mice used were approximately 27% for the curative test and 24% for the prophylactic test. Donor mice were sacrificed, and the blood was collected by cardiac puncture. Each experimental mouse was inoculated with 0.2 mL of blood suspension containing approximately 1 × 10^6^*P. berghei* ANKA intraperitoneally. For all experimental models of malaria (Figure 1), mice were randomized and divided into five groups (*n* = 5 animals per group). Piperine was administered orally at 10, 20, or 40 mg/kg bw. Body weight and temperature were measured at Day 0 and Day 4. The parasitaemia level was determined from Giemsa-stained blood films. Each mouse was monitored after treatment till their death. The survival rate and clinical signs were recorded.

### 2.3. Curative Test

The use of piperine to cure the early infection of *P. berghei* ANKA in mice was described by Riley and Peters [22]. Here, mice were inoculated with parasitized erythrocytes as mentioned above and left untreated until parasitaemia reached 2% or 72 h post inoculation. Groups I, II, and III were respectively given piperine 10, 20, and 40 mg/kg bw orally for four consecutive days. Artesunate 5 mg/kg bw (standard drug) was given to Group IV, and Group V (vehicle control) was given 0.2 mL DMSO. The percentage of parasitaemia and mean suppression of parasitaemia were calculated at Day 8.

### 2.4. Prophylactic Activity

The residual infection procedure first used by Peters was used to evaluate the prophylactic activity of piperine [23]. Piperine 10, 20, and 40 mg/kg BW were administered orally to Groups VI, VII, and VIII, respectively. Furthermore, Groups IX and *X* were treated with artesunate and DMSO, respectively. All treatments were given daily for four days. Then, all mice were infected with the *P. berghei* ANKA-parasitized erythrocytes on D5. The mean parasitaemia in each group was determined by microscopic examination of thin blood smears prepared from each mouse on D8 (72 h after infection).

### 2.5. Parasitaemia Determination

Thin blood smears were prepared on D8 for the curative test and the prophylactic activity. A drop of blood from the tail vein of the mouse was smeared on a glass slide and fixed with absolute methanol for 10 seconds. After fixation, the slides dried at room temperature for approximately 1 minute and were then stained with 10% Giemsa for 15 minutes. After washing with running water, the slides dried at room temperature for 1 minute. Parasitized red blood cells were counted using a binocular microscope (Optika B-190 TB, Italy) with oil immersion at 100x magnification. The percentage of parasitaemia was determined through the mean of ten visual fields and is calculated using the following formula:

(1) $$\begin{array}{c} \%\text{ parasitemia}=\frac{\text{number of parasitized RBC}}{\text{total number of RBC}}\;\times 100. \end{array}$$

Meanwhile, the percentage of suppression of the parasite is calculated using the following formula:

(2) $$\begin{array}{c} \%\text{ suppression}=\frac{\text{mean}\%\text{ parasitemia of untreated group}-\text{mean}\%\text{parasitemia of treated group}}{\text{mean}\%\text{parasitemia of untreated group}}\times 100. \end{array}$$

### 2.6. Determination of Clinical Signs

Every day after *P. berghei* ANKA infection, infected mice were routinely monitored (twice daily) for unusual symptoms (hunching, ruffled fur, limb paralysis, wobbly gait, convulsions, coma, and eventual mortality) [24]. Each sign was given a score of 1. Animals with severe clinical symptoms (cumulative scores ≥4) were sacrificed by cervical dislocation according to ethical guidelines, and the day of death was deemed to be the following day. Clinical symptoms were recorded for each experimental mouse.

### 2.7. Histopathological Examination

Organs were harvested and fixed with 10% formalin. Tissues were further processed through alcohol dehydration, followed by xylol clearing, and paraffin infiltration. Paraffin blocks were sectioned with a thickness of 3–4 µm, and the slides were deparaffinized in xylol and then stained with haematoxylin and eosin (all procedures were carried out at the Veterinary Pathology Laboratory, Faculty of Veterinary Medicine, Airlangga University). Slides were observed using a trinocular clinical light microscope with a digital camera (Olympus BX 53 with camera Olympus DP 23, Japan) connected to a computer (all procedures were carried out at the Vaccine Biology Institute, Army Medical Centre). Micrographs of the tissue were generated using the x10 objective lens for further analysis. Micrographs of the tissue from the control group were always compared with the treatment group. Changes were recorded using a standard nonlinear semiquantitative scoring system using a scale from 0 to 5, as previously described [25]. Significant findings were scored 0 for no change compared with usual morphology; a score of 1 denoted the least changes that could be detected by light microscopy (<10% of the affected tissue); a score of 2 was when the change was easily detectable but not a major hallmark (<20%); score 3 for more widespread changes that might be expected to associate with changes in organ function or weight; a score of 4 when almost 75% of the tissue was affected by the change; and score 5 for the entire tissue being impacted by changes that might be functionally relevant.

### 2.8. Ethical Approval

This research has been approved by the Research Ethics Committee, Padjadjaran University, Bandung, Indonesia (protocol number: 1045/UN.6. KEP/EC/2020). Experiments were carried out in accordance with laboratory animal care and use guidelines.

### 2.9. Statistical Analysis

The collected data were expressed as mean ± standard error of mean (SEM). Using GraphPad Prism version 8, the differences between means of measured parameters were compared using one-way analysis of variance (ANOVA) followed by Tukey's post hoc test. A *P* value < 0.05 was regarded as significant.

## 3. Results

### 3.1. Effect of Piperine on Body Weight

**Figure 2:**
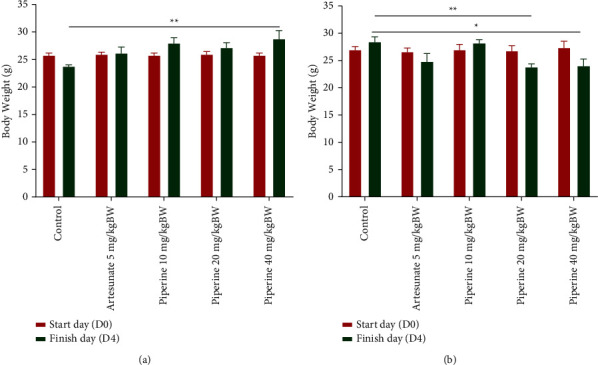
Effect of piperine administration on body weight of mice infected with *P. berghei* ANKA in the (a) curative test and (b) prophylactic test. Data are presented as mean ± SEM.  ^*∗∗*^*p* < 0.01;  ^*∗*^*p* < 0.05 compared with negative control (two-way ANOVA followed by Tukey's *post hoc* test).

In the curative test, all levels of piperine and artesunate prevented loss of body weight in mice compared with the negative control (Figure 2(a)). Both piperine-treated and artesunate-treated mice gained some of their body weight. Nevertheless, the piperine-treated mice had better body weight status than artesunate-treated mice. In the prophylactic test, the administration of piperine 20 mg/kg bw and 40 mg/kg bw had a significant effect (*p* < 0.05) on weight loss compared with the control group (Figure 2(b)).

### 3.2. Effect of Piperine on Body Temperature

**Figure 3:**
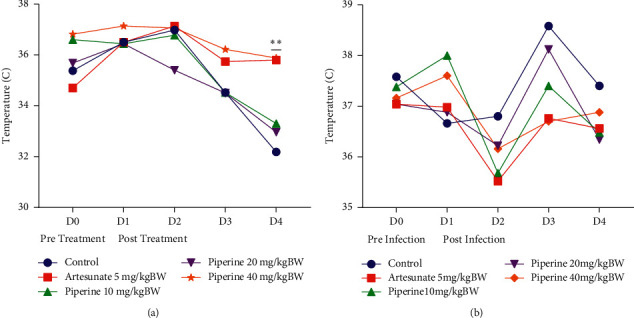
Effect of piperine administration on rectal temperature of mice infected with *P. berghei* ANKA in (a) curative test and (b) prophylactic test. Data are presented as mean ± SEM.  ^*∗∗*^*p* < 0.01 compared with negative control (two-way ANOVA followed by Tukey's *post hoc* test).

In the curative test, administration of piperine 40 mg/kg bw and artesunate 5 mg/kg bw prevented reduction in rectal temperature compared with the negative control (Figure 3(a)). Meanwhile, in the prophylactic model, administration of piperine did not prevent rectal temperature reduction after infection by *P. berghei* ANKA (Figure 3(b)).

### 3.3. Effect of Piperine on Parasitaemia

**Figure 4:**
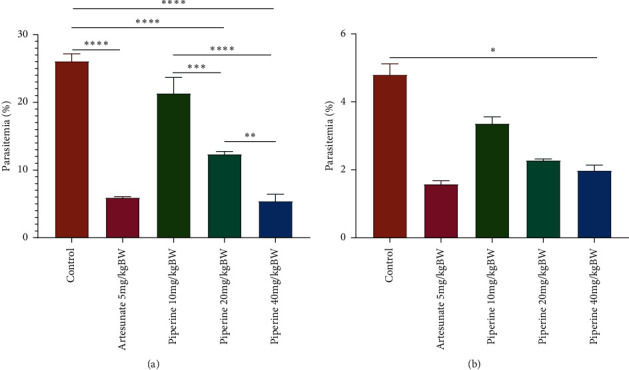
Degree of parasitaemia after piperine administration in mice infected with *P. berghei* ANKA in the (a) curative test and (b) prophylactic test (*n* = 5 per group). Data are presented as mean ± SEM.  ^*∗∗∗∗*^*p* < 0.0001,  ^*∗∗∗*^*p* < 0.001,  ^*∗∗*^*p* < 0.01 compared with negative control (one-way ANOVA followed by Tukey's *post hoc* test).

The curative and prophylactic activities of piperine were evaluated at three doses (10, 20, and 40 mg/kg bw). In a curative test (Figure 4(a))), two doses (20 and 40 mg/kg bw) demonstrated significant parasitaemia suppression (52.61% and 79.21%) compared with the control group (*p* < 0.05). In the prophylactic test, piperine demonstrated prophylactic activity resulting in significant (*p* < 0.05) parasitaemia suppression at 10, 20, and 40 mg/kg bw when compared with the control mice, with chemosuppression rates of 30.0%, 56.7%, and 58.8%, respectively (Figure 4(b)). Comparison among dose levels between the curative and prophylactic tests indicated that the 40 mg/kg bw dose had significant parasitaemia suppression activity when compared with the 10 and 20 mg/kg bw doses. However, the effectiveness of the 40 mg/kg bw dose was not significantly different from the standard drug (artesunate 5 mg/kg bw).

### 3.4. Effect of Piperine on Clinical Signs

**Figure 5:**
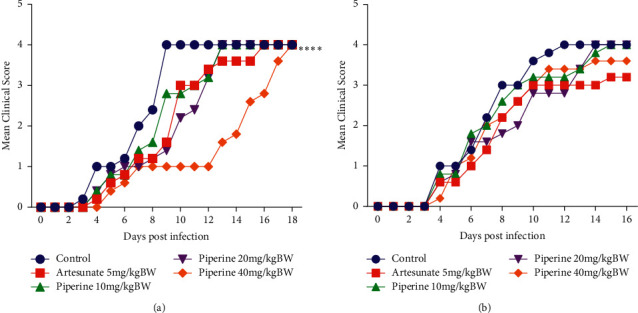
Effect of piperine administration on the clinical score of mice infected with *P. berghei* ANKA in the (a) curative test and (b) prophylactic test. Data are presented as mean ± SEM.  ^*∗∗∗∗*^*p* < 0.0001 compared with negative control (two-way ANOVA followed by Tukey's *post hoc* test).

In the curative test, the clinical course of mice receiving piperine 20 mg/kg bw or 40 mg/kg bw was significantly reduced on the ninth (D8) and tenth (D9) days compared with the negative control (both *p* < 0.05). The decrease in clinical signs in the group treated with piperine 40 mg/kg bw was still observed until Day 14 (Figure 5(a)). Meanwhile, in the prophylactic test, the clinical symptoms were not significantly different compared with the negative control (Figure 5(b)).

### 3.5. Effect of Piperine on Survival Rate

**Figure 6:**
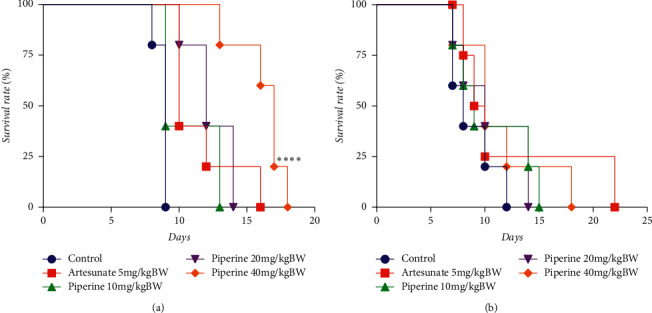
Survival rate after piperine administration in mice infected with *P. berghei* ANKA in the (a) curative test and (b) prophylactic test.  ^*∗∗∗∗*^*p* < 0.0001 compared with negative control (Kaplan–Meier survival curve).

In the curative test, the survival times were prolonged only in the group treated with 40 mg/kg bw of piperine (Figure 6(a)). In contrast, in the prophylactic test, all doses of piperine failed to induce significant prolongation of survival time when compared with the vehicle control (Figure 6(b)).

### 3.6. Effect of Piperine on Histopathological Examination

Histopathological studies revealed some of the damages caused by the malaria parasite and the level of reduction of these damages by the administered drugs. Particularly, excess fluid in the lungs (pulmonary edema) and thickening of the alveolar wall in the lungs (Table 1), death of liver cells (hepatic necrosis) and also Kupffer cell hyperplasia (Table 2), inflammation of the spleen (splenitis) (Table 3), inflammation of the glomeruli (glomerulonephritis) (Table 4) are the damages. All these damages were severe in the control group. In the curative test, the effect of piperine 40 mg/kg bw on reducing the pathologies caused by malaria parasites was more noticeable compared with the control group and another dose of piperine. However, it was not significantly different from the standard drug artesunate.

## 4. Discussion

Malaria remains one of the most troubling parasitic diseases, affecting all developing countries and causing a serious financial catastrophe. The situation is aggravated by the unavailability of consistent drugs targeting malaria (especially against resistant parasites) and the lack of malaria vaccines for control and prevention [14]. To overcome the challenge of resistance to available antimalarial agents, medicinal plants are believed to be a key source in the search for active components that have better efficacy [15]. More than 1200 medicinal plants have been used worldwide to treat malaria, one of which is *Piper nigrum* [26]. Piperine is one of the main active components isolated from *P. nigrum*. It is responsible for a range of biological activities, including anti-inflammatory, antioxidant, and antiplasmodial activity *in vitro* [17, 20, 27–29]. In this context, this study attempted to assess the curative and prophylactic capability of piperine for established malaria infection. In both assays presented here, the evaluation of the percentage of inhibition of parasitaemia is the most reliable parameter. Additionally, the clinical signs and the alteration caused by piperine on histopathological parameters were assessed after treatment.

In the curative test on established malaria infection, piperine showed a significant parasitaemia chemosuppression, with a maximum of 79.21% in the dose of 40 mg/kg bw (Figure 4(a)). The longest mean survival time for the mice was strongly associated with the maximum parasitaemia inhibition; this was in line with another *in vivo* antimalarial test [30, 31].

*Plasmodium* infection in erythrocytes causes clinical symptoms in humans and animal models. Evaluation of clinical signs provides enormous information related to the pathogenesis of a disease. Clinical manifestations including slouching, ruffling of the feathers, unsteady gait, paralysis of the limbs, convulsions, and subsequent death were observed and often reflect high levels of parasitaemia [32]. A proportion of infected subjects developed neurological symptoms that rapidly progressed to death within 5 to 10 days of infection [33]. The result of this study revealed that administration of piperine (both at 20 mg/kg bw and 40 mg/kg bw) significantly reduced clinical signs on the ninth (D8) and tenth (D9) days compared with animals that were given vehicle. The decrease in clinical signs in the piperine 40 mg/kg bw group was still observed until Day 14 (Figure 5(a)). The ability of piperine to inhibit the progression of parasitaemia, reduce clinical symptoms, and prolong the survival of animals was presumably due to it acting on the erythrocytic phase, specifically changing the morphology of infected red blood cells. At the late ring to trophozoites stages, the cytoplasm of infected red blood cells condenses, rendering them defective [28, 34].

The pathogenesis of malaria can be severe, causing complications and disorders in various organ systems of the body (brain, lungs, liver, kidneys, and spleen) [35]. Malaria caused by *P. berghei* can infect red blood cells, causing microvascular obstruction due to parasite sequestration [35–37]. The increase in free radicals provoked by malaria infection causes cell membrane lipid peroxidation, which triggers an inflammatory response and pathological changes in the lung [35, 38]. The treatment of *P. berghei* infection with piperine and artesunate caused a decrease in lung damage (Supplementary Figure (s1)), namely the appearance of hemorrhage, alveolar congestion, edema, and thickening of the alveolar septa. This occurs because of barriers to the formation of reactive oxygen species. In the treatment of malaria, it is important to use ingredients with high antioxidant properties since antioxidants play a role in supporting the immune system and reducing oxidative stress. Piperine is a powerful antioxidant that can reduce lung damage [39–41]; the strong antioxidant potential of piperine has been reported in an experimental lung cancer model [16, 42].

Several previous studies have shown that liver damage can occur during the erythrocytic stage in the malaria parasite's life cycle [43]. It is known that several inflammatory stimuli, including immune responses to infectious agents, can cause liver injury [43–45]. Pathogens such as *P. berghei* are also known to induce acute inflammation in the liver [43, 46]. In this study, the livers of mice infected with *P. berghei* ANKA showed the presence of macrophages, had inflammatory cell infiltration in the sinusoids, changes in the infiltration of lymphocytes, and polymorphonuclear cells in the portal hepatocyte, and central venous congestion. Interestingly, the piperine- and artesunate-treated groups exhibited mild inflammation and the absence of macrophages (Supplementary Figure s2). The absence of macrophages may be due to a decreased proinflammatory immune response, as shown in previous studies [45, 47, 48].

The malaria parasite is considered a foreign body when it enters the host and is immediately recognized by a specific immune response. This causes the proliferation of the splenic white pulp (lymphocytes, macrophages, and reticular cells increase in size) so that the diameter of the splenic white pulp also increases [49]. Consequently, the spleen in organisms infected with malaria will enlarge as a result of increased erythropoiesis and hematopoiesis [50]. The administration of piperine and artesunate is known to reduce the diameter of the splenic white pulp (Supplementary Figure (s3)). This is in accordance with previous studies, which explained that piperine contains antioxidants that can improve the host's immune system through increased phagocytic cell activity [35, 50]. This has an impact on reducing the number of *Plasmodium* parasites, reducing the inflammation that occurs in malaria-infected spleen, thus reducing cell swelling [35].

The kidney morphology showed tubular necrosis and inflammation, which is associated with *P. berghei* infection in mice increasing proinflammatory molecules and oxidative stress [36]. In complex infectious conditions, loss of renal endothelial integrity is correlated with changes in oxygen and reactive nitrogen species, heme toxic, and proinflammatory molecules, leading to reduced oxygen levels to cells and tissues. This condition triggers the occurrence of a hypoxic microenvironment, decreased renal perfusion, acute tubular necrosis, and decreased cellular defence mechanisms, impacting the event of acute kidney failure [36, 51]. In the present study, piperine administration reduced all tissue injury caused by *P. berghei* ANKA infection (Supplementary Figure (s4)) associated with reduced inflammation.

Some traditional plants that showed antiplasmodial activity in curative tests also demonstrated prophylactic activity against *P. berghei* ANKA with minimal effects [52]. Therefore, the present study also aligns with the previous research on another traditional plant against *P. berghei* [53].

The prophylactic effect of piperine showed antiplasmodial activity in *P. berghei* ANKA*-*infected mice with parasitaemia chemosuppression of 58.8% at a dose of 40 mg/kg bw. In contrast to the curative test, piperine 40 mg/kg bw had a lower percentage of parasitaemia inhibition when compared with artesunate 5 mg/kg bw. Furthermore, the administration of piperine in a dose-dependent manner as prophylaxis did not show different results for rectal temperature and clinical scores in *P. berghei* ANKA-infected mice. This suggests that piperine in curative trials reduces the severity of clinical symptoms after *P. berghei* ANKA infection but does not decrease the severity of infection. This phenomenon may be caused by a wash-out of piperine levels so that, at D5, piperine is no longer in the blood. Previous studies have shown that the half-life of oral piperine is 1,224 hours with a total body clearance of 2,656 L/kg/hour. Furthermore, the peak plasma concentration of piperine in plasma after oral administration was found to be 0.983 g/mL, occurring approximately 2 h post dose [54]. Another study conducted on rats reported that only about 0.1%–0.25% of piperine administered orally could be detected in the liver [55]. The administration of piperine 20 mg/kg bw and 40 mg/kg bw in the prophylactic tests caused weight loss. This may occur because piperine is given for 4 days before infection so that piperine levels in the blood decrease and are unable to fight the disease.

The mechanism of action of piperine as an antimalarial is still unclear. Previously, the antimalarial activity of piperine (*in vivo*) was carried out by combining piperine and curcumin in a mouse model infected with *Plasmodium chabaudi.* Oral administration of piperine 20 mg/kg bw/day for 15 days was used as an enhancer to increase the bioavailability of curcumin (300 mg/kg bw/day), which exhibits moderate antimalarial activity [30]. In another *in vivo* study, a combination of piperine-curcumin-chloroquine (20mg-250mg-2.5 mg) administered for 4 days and followed up for 7 days produced an additive impact in reducing parasitic load (7 days after treatment). This combination was shown to be effective in reducing parasitaemia by up to 45% in chloroquine-resistant AS-3CQ *P. chabaudi*-infected mice [31]. Piperine (1-piperoyl piperidine) is a nitrogen-containing alkaloid present in the fruit of black pepper (*Piper nigrum*), and other *Piper* species [40]. Alkaloids over the years have been recognized as important phytoconstituents with interesting biological properties [58]. Hence, it can be hypothesized that piperine has parasite-killing potential as demonstrated by other alkaloids, such as quinine and chloroquine. The mechanism of action of quinine is similar to chloroquine (acts as a blood schizonticide) [56]. Quinine is known to inhibit the hexokinase activity of *Plasmodium berghei* [57], while chloroquine acts by inhibiting heme polymerization in parasites [59]. However, further investigations to elucidate the mechanism of action of piperine at the cellular and molecular levels should be carried out. Although this murine model has some limitations, it served to successfully validate the significant inhibitory activity of piperine in *P. berghei*-infected mice.

## 5. Conclusion

The administration of piperine (40 mg/kg bw) was comparable to artesunate (5 mg/kg bw) in the curative test on *P. berghei* ANKA-infected mice, which may be mediated by the antiparasitic and anti-inflammatory properties of piperine. Therefore, piperine is a promising candidate for further development as an antimalarial drug or adjunct therapy in combination with other drugs.

## Figures and Tables

**Table 1:** Semiquantitative analysis of lung histopathology in the curative test.

Group	Histopathological parameters			
	Hemorrhage	Alveolar congestion	Edema	Thickening septa alveoli
Control	3.00 ± 0.00	3.00 ± 0.00	3.50 ± 0.50	3.50 ± 0.50
Artesunate 5 mg/kg bw	0.50 ± 0.50	0.50 ± 0.50	0.50 ± 0.50	0.50 ± 0.50
Piperine 10 mg/kg bw	1.50 ± 0.50	1.50 ± 0.50	2.50 ± 0.50	2.50 ± 0.50
Piperine 20 mg/kg bw	1.50 ± 0.50	0.50 ± 0.50	1.00 ± 0.00	1.50 ± 0.50
Piperine 40 mg/kg bw	0.00 ± 0.00	0.50 ± 0.50	0.50 ± 0.50	0.50 ± 0.50

**Table 2:** Semiquantitative analysis of liver histopathology in the curative test.

Group	Histopathological parameters			
	Hyperplasia Kupffer cells	Sinusoid congestion	Portal inflammation	Necrosis
Control	3.50 ± 0.50	3.50 ± 0.50	3.50 ± 0.50	4.00 ± 0.00
Artesunate 5 mg/kg bw	1.00 ± 0.00	1.00 ± 0.00	1.00 ± 0.00	1.00 ± 0.00
Piperine 10 mg/kg bw	3.50 ± 0.50	2.00 ± 0.00	2.00 ± 0.00	3.00 ± 0.00
Piperine 20 mg/kg bw	2.50 ± 0.50	1.00 ± 0.00	1.50 ± 0.50	2.50 ± 0.50
Piperine 40 mg/kg bw	1.00 ± 0.00	1.00 ± 0.00	1.00 ± 0.00	1.00 ± 0.00

**Table 3:** Semiquantitative analysis of spleen histopathology in the curative test.

Group	Histopathological parameters			
	Hemorrhage	Lymphocyte depletion	Inflammation	Necrosis
Control	1.00 ± 0.00	2.00 ± 0.00	1.00 ± 0.00	3.00 ± 0.00
Artesunate 5 mg/kg bw	0.50 ± 0.50	0.50 ± 0.50	0.50 ± 0.50	0.50 ± 0.50
Piperine 10 mg/kg bw	1.00 ± 0.00	1.00 ± 0.00	1.00 ± 0.00	1.00 ± 0.00
Piperine 20 mg/kg bw	1.00 ± 0.00	1.00 ± 0.00	0.50 ± 0.50	1.00 ± 0.00
Piperine 40 mg/kg bw	0.00 ± 0.00	0.50 ± 0.50	1.00 ± 0.00	0.50 ± 0.50

**Table 4:** Semiquantitative analysis of kidney histopathology in the curative test.

Group	Histopathological parameters			
	Tubular necrosis	Glomerulonephritis	Nephritis interstitials	Congestion
Control	3.00 ± 0.00	4.00 ± 0.00	2.50 ± 0.50	2.00 ± 0.00
Artesunate 5 mg/kg bw	0.50 ± 0.50	0.50 ± 0.50	0.50 ± 0.50	0.50 ± 0.50
Piperine 10 mg/kg bw	1.50 ± 0.00	2.50 ± 0.00	1.50 ± 0.50	1.00 ± 0.00
Piperine 20 mg/kg bw	1.00 ± 0.00	2.00 ± 0.50	1.50 ± 0.50	0.50 ± 0.50
Piperine 40 mg/kg bw	0.50 ± 0.50	0.50 ± 0.50	0.50 ± 0.50	0.00 ± 0.00

## Data Availability

The data used to support the findings of this study are included within the article.

## Supplementary Materials

Supplementary Materials Figure S1: histopathology of lung, Figure S2: histopathology of liver, Figure S3: histopathology of spleen, and Figure S4: histopathology of kidney.
