# Supplementary material for: Oral Administration of Piperine as Curative and Prophylaxis Reduces Parasitaemia in Plasmodium berghei ANKA-Infected Mice
Source: J Trop Med. 2022 Mar 22;2022:5721449. doi: 10.1155/2022/5721449 (PMC8964209; doi:10.1155/2022/5721449)
Supplement: Supplementary Materials — Figure S1: histopathology of lung, Figure S2: histopathology of liver, Figure S3: histopathology of spleen, and Figure S4: histopathology of kidney. [file 5721449.f1.zip › 5721449.f1/Supplementary Figure S2. Histopathology of Liver.docx]

**Supplementary Figure S2. Histopathology of Liver**


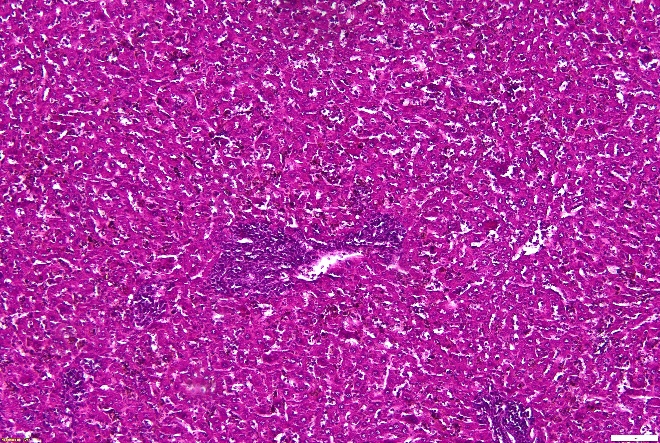

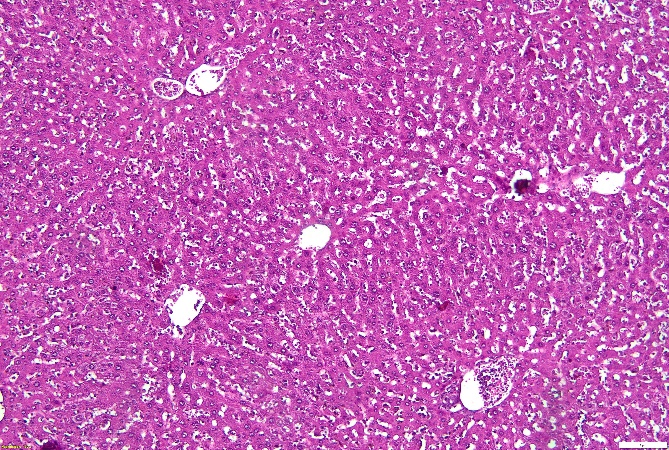


Control (DMSO)

Artesunate 5 mg/kg BW

Piperine 10 mg/kg BW

Piperine 20 mg/kg BW

Piperine 40 mg/kg BW

**A**

**B**

**C**

**D**

**E**


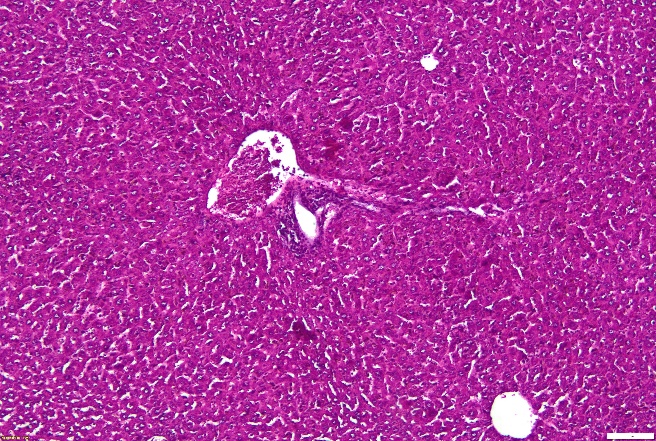

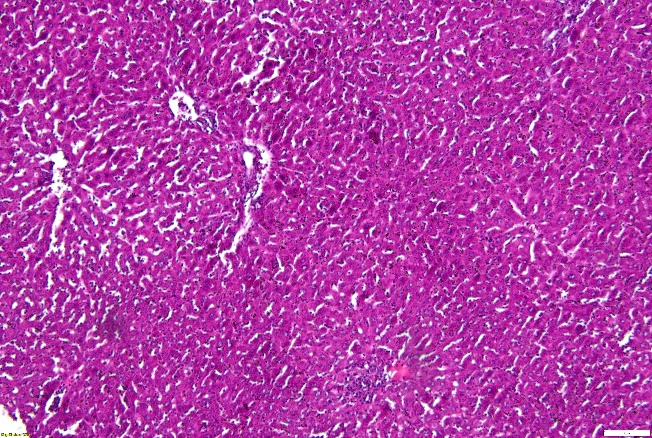


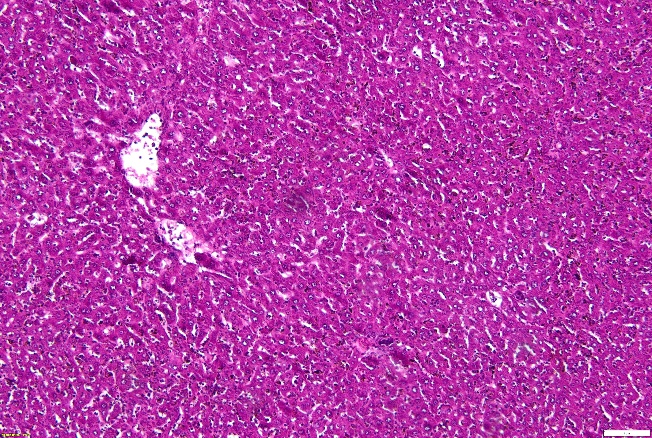


Photomicrographs of H&E staining of livers of animal treated with piperine (10, 20, and 40mg/kg bw), artesunate (5 mg/kg bw), or DMSO in curative test, showing some Kupffer cells in the hepatocytes, inflammation, and *P. berghei*-infected red cells in the lumen of blood vessels. Low magnification (10x).
